# Psilocin suppresses methamphetamine‐induced hyperlocomotion and acquisition of conditioned place preference via D2R‐mediated ERK signaling

**DOI:** 10.1111/cns.14054

**Published:** 2023-01-10

**Authors:** Jing Wang, Min Liang, Qing Shang, Hongyan Qian, Ran An, Hua Liu, Gaojie Shao, Tao Li, Xinshe Liu

**Affiliations:** ^1^ College of Forensic Medicine Xi'an Jiaotong University Health Science Center Xi'an China; ^2^ Institute of Forensic Injury, Institute of Forensic Bioevidence, Western China Science and Technology Innovation Harbor Xi'an Jiaotong University Xi'an China; ^3^ Key Laboratory of Forensic Toxicology Beijing China

**Keywords:** conditioned place preference, D2R, METH‐induced hyperlocomotion, p‐ERK, psilocin

## Abstract

**Aim:**

Psilocin is an active metabolite form of psilocybin and exerts psychoactive effects. Recent studies suggest that psilocin may have regulatory effects on abuse drugs, but the mechanisms remain unclear. In this study, we want to explore the effects of psilocin on methamphetamine (METH)‐induced alterations of behavior in mice and its molecular mechanisms.

**Methods:**

Acute METH administration model and conditioned place preference (CPP) model were used to investigate the effects of psilocin on METH‐induced alterations of behavior. Western blot was used to detect the expression of proteins.

**Results:**

In the acute 2 mg/kg METH administration model, 1 mg/kg psilocin counteracted METH‐induced elevation of activity. In the 1 mg/kg METH‐induced CPP model, 1 mg/kg psilocin inhibited CPP formation during the acquisition phase. However, psilocin did not impact METH extinction and relapse. Molecular results showed that the regulatory effect of psilocin on METH was underscored by altered expression of dopamine 2 receptor (D2R) and phosphorylated extra‐cellular signal‐regulated kinase (p‐ERK) in the prefrontal cortex (PFC), nucleus accumbens (NAc), and ventral tegmental area (VTA). Trifluoperazine (TFP)‐2HCl is a D2R inhibitor, and SCH772984 is a selective extra‐cellular signal‐regulated kinase (ERK) inhibitor that effectively inhibits ERK1/2 phosphorylation. The results indicated that 2 mg/kg TFP‐2HCl and 10 mg/kg SCH772984 blocked METH‐induced hyperactivity and acquisition of METH‐induced CPP.

**Conclusion:**

Psilocin has regulatory effects on METH‐induced alterations of behavior in mice via D2R‐mediated signal regulation of ERK phosphorylation.

## Graphical abstract

Psilocin has regulatory effects on METH‐induced alterations of behavior in mice via D2R‐mediated signal regulation of ERK phosphorylation indicating that psilocin may have the potential treatment in METH.

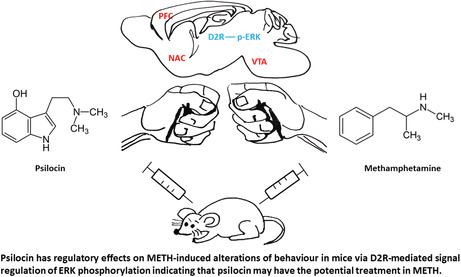

## 1 INTRODUCTION

Psilocybin is an indole alkylamine extracted from hallucinogenic mushrooms. Psilocin (4‐hydroxy‐dimethyltryptamine) is a dephosphorylation production of psilocybin that easily crosses the blood–brain barrier and exerts psychoactive effects.^1^ A growing body of research suggests that psilocin influences cognition and emotion in both animals and humans, without affecting psychomotor stimulation, alertness, attentiveness, memory, or orientation.^2^ Moreover, clinical research suggests that psilocin may be safe and effective for treating neuropsychiatric disorders, including depressive disorder, cancer‐related anxiety,^3^ and alcohol^4^ and tobacco addiction.^5^ Psilocin is a postsynaptic 5‐HTR agonist that predominantly targets the 5‐HT2AR subtype.^6^ In this regard, 5‐HTR agonists induce‐allosteric inhibition of dopamine receptor 2 (D2R) signaling, and hallucinogenic 5‐HT2AR agonists increase the Bmax values of D2R antagonist binding sites.^7^ Moreover, ic3 (the third intracellular loop of D2R containing adjacent arginine residues) and C‐tail (the acidic glutamate residues of 5‐HT2AR) play a key role in the dimerization of 5‐HT2AR and D2R.^8^ Vollenweider et al.^9^ examined the binding of ^[11C]^raclopride to D2R in healthy volunteers after placebo and psilocybin treatment and reported an increase in D2R occupancy by endogenous dopamine.

Methamphetamine (METH) is a neurotoxic psychostimulant. More than 35 million people use amphetamine‐type substances worldwide.^10^ A recent study has indicated that the D2R plays a key regulatory role in the molecular and behavioral responses to drug abuse.^11^ Acute METH induces psychostimulant effects including euphoria, alertness, and increased locomotor activity. Moreover, acute METH‐induced psychotropic effects may contribute to the augmentation of subsequent drug‐seeking and drug‐taking behavior.^12^ These effects are induced by the stimulation of neurotransmitter release via actions on the dopamine transporter (DAT), resulting in increased dopamine (DA) release. Indeed, acute METH increases D2R expression associated with activation of the mesolimbic dopaminergic pathway.^13^ However, positron emission tomography and magnetic resonance imaging data in human indicate that persist beyond the period of METH consumption reduced the density of D2 receptor. The mesolimbic dopaminergic reward‐pathway is the main neural pathway that regulates the reinforcing effects of addictive drugs and comprises DA neurons in the ventral tegmental area (VTA) that project to the nucleus accumbens (NAc)^14^ and prefrontal cortex (PFC).^15^

Research suggests that altered striatal DA‐dependent behaviors are caused by elevated D2R expression and upregulated extracellular signal‐regulated kinase (ERK) 1/2 activation,^16^ and D2R over‐activation leads to a decrease protein kinase B (AKT) phosphorylation in cortical neurons, eventually leading to neurite lesions.^17^ Recent studies have suggested that ERK signaling may constitute a common pathway to different drugs of abuse, leading to the addictive state.^18^ Chronic administration of METH in rats causes behavioral and molecular changes, including neurodegenerative effects that may be partially mediated by inhibition of phosphorylated protein kinase B (p‐AKT).^19^ In addition, D2R activation is modulated by ERK^20^ and AKT phosphorylation levels.^21^

Based on recent studies, we hypothesized that psilocin might have an effect on METH‐induced alterations of behavior via the D2R‐mediated signal regulation of ERK phosphorylation or AKT phosphorylation. Therefore, this study investigated the levels of D2R, p‐AKT, AKT, phosphorylated ERK (p‐ERK), and ERK expression in the PFC, NAc, and VTA in order to explore the molecular mechanisms of the effect of psilocin on METH‐induced alterations of behavior in mice.

## 2 MATERIALS AND METHODS

### 2.1 Animals

In total, 204 8‐week‐old C57BL/6J male mice were purchased from Beijing Vital River Laboratory Animal Technology. Mice were housed in four mice per cage with water and standard chow available ad libitum in a controlled environment (temperature, 20°C–24°C; humidity, 40%–60%; 12/12 h light/dark cycle). Prior to experimentation, all animals were housed for 1 week to adapt to the environment to avoid the confounding effects of environmental factors on behavior and protein expression. Mice were fed and housed according to the Guidelines for the Care and Use of Laboratory Animals issued by the National Institutes of Health, USA. Ethical approval for this research was obtained from the Institutional Animal Care and Use Committee of Xi'an Jiaotong University.

### 2.2 Drugs

Methamphetamine hydrochloride (China Pharmaceutical and Biological Products, 99.9%) and Psilocin (Cayman Chemical Company) were dissolved in 0.9% saline. TFP‐2HCl (TargetMol Chemicals Inc) and SCH772984 (MedChemExpress) were dissolved in 5% dimethyl sulfoxide (DMSO) solution. In the acute METH administration model, the dose of METH was 2 mg/kg; the doses of psilocin were 5 mg/kg, 1 mg/kg, or 0.25 mg/kg; the dose of TFP‐2HCl was 2 mg/kg, and the dose of SCH772984 was 10 mg/kg. In the METH‐induced CPP model, the dose of METH, psilocin, TFP‐2HCl, and SCH772984 were 1, 1, 2, and 10 mg/kg, respectively.

### 2.3 Acute METH administration model

In the first part of the experiment, 24 mice were divided into four groups including control, 5, 1, and 0.25 mg/kg psilocin. In the second part of the experiment, 25 mice were divided into four groups including control, METH only, and, 1 or 0.25 mg/kg psilocin administered prior to i.p injection of METH. In the third part of the experiment, 36 mice were divided into six groups including control, 2 mg/kg TFP‐2HCl, 10 mg/kg SCH772984, METH, 2 mg/kg TFP‐2HCl and 10 mg/kg SCH772984 administered prior to i.p. injection of METH. In all three parts of the experiment, mouse activity was monitored in an open‐field for 1 h. At the end of the experimentation, mice were sacrificed and the PFC, NAc and VTA were collected. Samples were stored at −80°C until subsequent use.

### 2.4 CPP model

Four CPP apparatuses each consisting of two equal compartments (15 cm × 15 cm × 37 cm) were used in this study. One was a white compartment with a metal grid floor and the other was a black compartment with a metal mesh floor. A movable baffle was in the center of apparatus. During the test period, the mice was allowed to shuttle freely in the apparatus by opening the baffle, and during training period, the mice was restricted to one side of the apparatus by closing the baffle. In this study, pre‐test results showed that mice spent more time in the black compartment than in the white compartment. Therefore, the black compartment was the “preferred‐one” and the white compartment was the “non‐preferred‐one”.

On day1, mice were pre‐tested for 15 min. Mice with abnormal activity (low motor activity, <20 shuttles, or more than 600 s spent on either side) were excluded.

In the acquisition phase (days 2–9), on days 2, 4, 6, and 8, all groups were injected with saline and placed in the black compartment for 45 min. On days 3, 5, 7, and 9, the control group was injected with saline; the drug group was injected with 1 mg/kg psilocin, 2 mg/kg TFP‐2HCl, or 10 mg/kg SCH772984; the METH group was injected with METH; and the METH with drug intervention group was injected with 1 mg/kg psilocin, 2 mg/kg TFP‐2HCl, or 10 mg/kg SCH772984 30 min before METH administration. All groups were placed in the white compartment for 45 min. At post‐test (day 10), all mice were allowed to explore the two compartments freely for 15 min.

In the extinction phase (days 11–22), four groups including control, PI, METH‐Ext1, and METH‐Ext2 were subjected to extinction training and testing. The METH group with CPP was divided into METH‐Ext1 and METH‐Ext2 groups. On day 11, four groups were injected with saline and placed in the black compartment for 45 min. On day 12, the control and METH‐Ext1 groups were injected with saline; PI and METH‐Ext2 groups were injected with psilocin, and all groups were placed in the white compartment for 45 min. On day 13, all mice were allowed to explore the two compartments freely for 15 min. This procedure was repeated three times over days 14–22.

In the reinstatement phase, four groups including Control, PI, METH‐Rein1 and METH‐Rein2 were tested. The METH‐Ext1 group was divided into METH‐Rein1 and METH‐Rein2 groups. The METH‐Rein1 group was injected (i.p) with METH, and the METH‐Rein2 group was injected (i.p) with 1 mg/kg psilocin prior to METH injections. The mice were then allowed to explore the two compartments freely for 15 min. At the end of the experimentation, the mice were sacrificed, and the PFC, NAc, and VTA were collected. Samples were stored at −80°C until subsequent use.

### 2.5 Protein extraction

Samples were homogenized in RIPA buffer with protease inhibitor (PMSF). Proteins were extracted with a high‐throughput tissue grinder (SCIENTZ‐48). Subsequently, proteins were centrifuged at 12,000 *g* for 15 min at 4°C. Protein concentration was measured using a BCA assay. Loading buffer was added to the samples, and samples were boiled at 100°C for 5 min.

### 2.6 Western blotting

The boiled protein samples were separated by 12% SDS‐PAGE (15 μg per lane). The proteins were transferred to a polyvinylidene fluoride (PVDF) membrane, and 5% skim milk was used to block the membrane at room temperature for 4 h. Next, the PVDF membrane was incubated with anti‐D2R (Abcam), anti‐β‐actin (Cell Signaling Technology), anti‐AKT (Cell Signaling Technology), anti‐p‐AKT (Thr308, Cell Signaling Technology), anti‐ERK (Cell Signaling Technology), and anti‐p‐ERK (Thr202/Tyr204, Cell Signaling Technology) antibodies at 4°C overnight. The next day, the membrane was washed with phosphate‐buffered saline with Tween‐20 (PBST) three times 10 min per wash and incubated with secondary antibodies (1:10,000) at room temperature for 2 h. The membrane was washed with PBST three times 10 min per wash, after which an enhanced chemiluminescence (ECL) kit (Millipore Corporation) was used to detect the proteins. The signal intensities of the bands were analyzed using ImageJ software (NIH). Each band was normalized against β‐actin for analysis.

### 2.7 Statistical analysis

GraphPad Prism (version 8.0) was used for all statistical analyses. All data are presented as means ± SEM. Bartlett's test for homogeneity of variance was used to ensure a normal distribution of data and all data were normally distributed. Acquisition and extinction of METH‐induced CPP data were analyzed using two‐way ANOVA. The effects of different concentrations of psilocin, acute METH administration model, reinstatement of METH‐induced CPP, and western blot data were analyzed using one‐way ANOVA. All post hoc pairwise comparisons were performed using the Bonferroni test. *p*‐values < 0.05 were considered statistically significant, with *and ^#^, ** and ^##^, *** and ^###^ indicating *p* < 0.05, *p* < 0.01, and *p* < 0.001, respectively.

## 3 RESULTS

### 3.1 Effects of different psilocin concentrations

**FIGURE 1:**
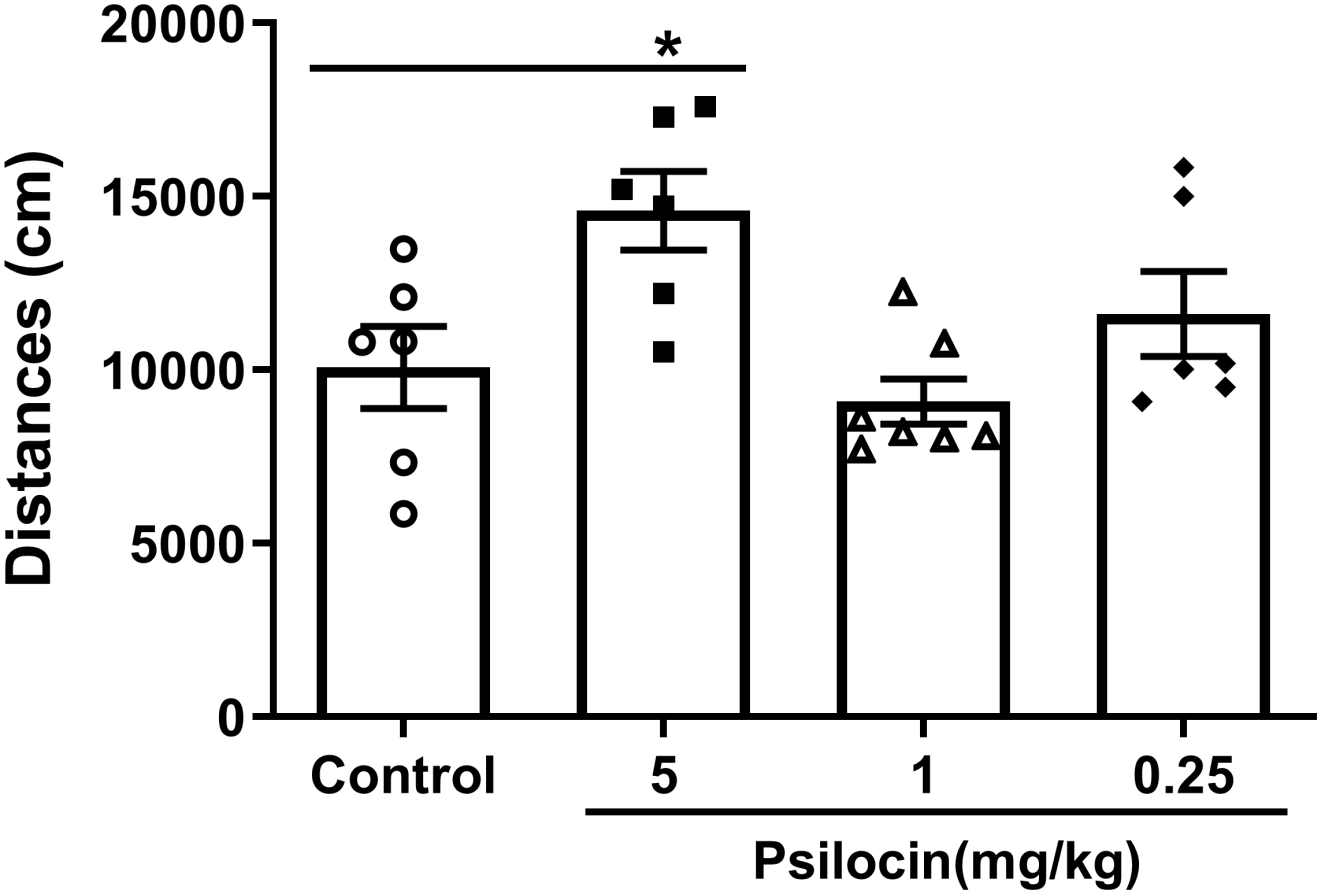
Effects of different concentrations of psilocin on locomotor activity in mice. Only the 5 mg/kg psilocin group increased mice activity comparing with the control group. Data are presented as the mean ± SEM (*n* = 6–7). *p < 0.05 versus the control group

Three different concentrations of psilocin 5, 1, and 0.25 mg/kg were injected into mice. Significant differences were observed between the control group and three concentrations of psilocin groups (*F*_(3,21)_ = 5.300, *p* = 0.007), whereby the 5 mg/kg psilocin group exhibited increased activity compared to the control group (*p* < 0.05). However, the activity of 1 and 0.25 mg/kg psilocin groups was not significantly different from that of the control group (Figure 1).

### 3.2 Regulatory effects of psilocin on acute METH administration model

**FIGURE 2:**
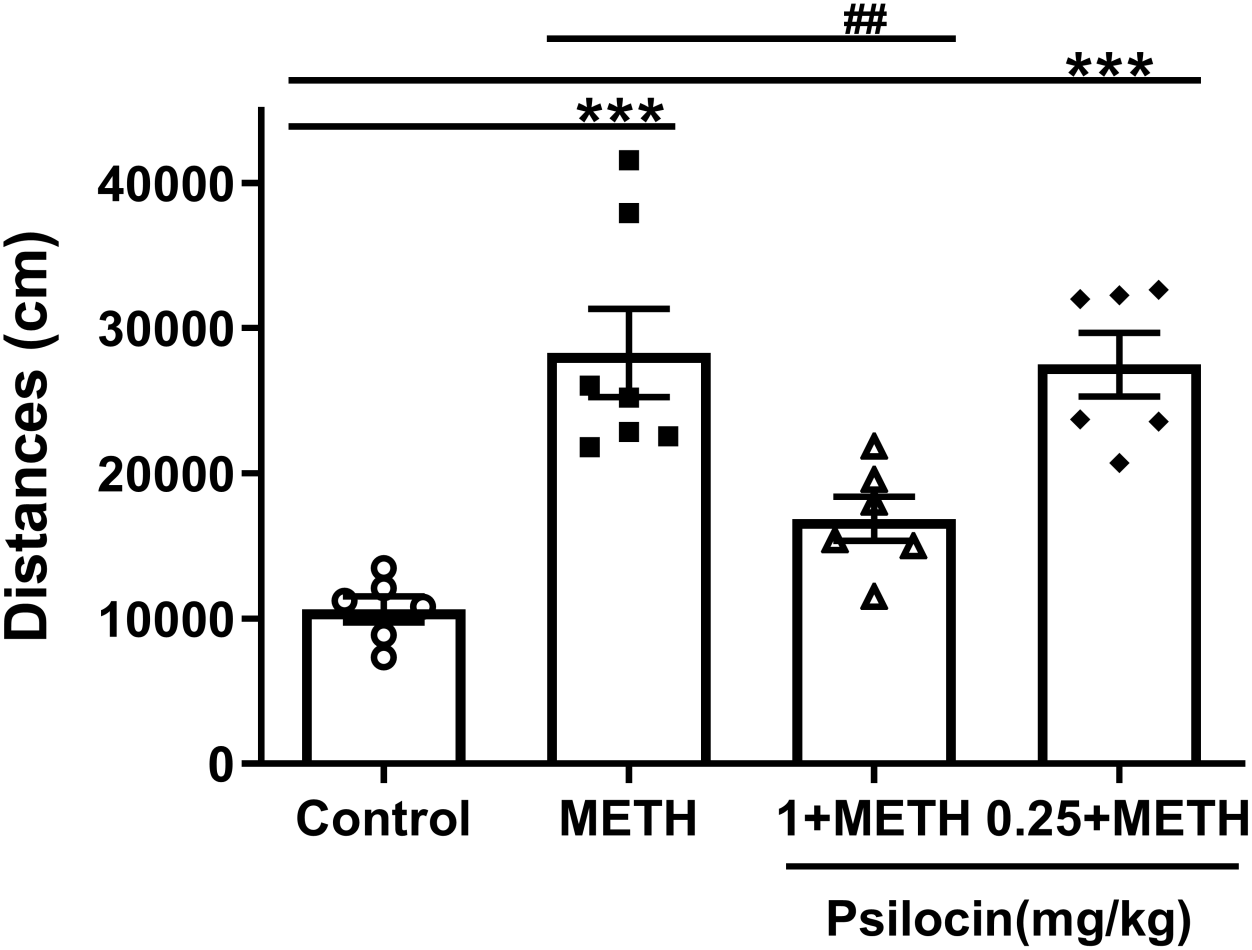
Effects of different concentrations of psilocin on hyperlocomotion induced by acute METH. 1 mg/kg psilocin group could inhibit METH‐induced hyperactivity. Data are presented as the mean ± SEM (*n* = 6–7). ***p < 0.001 vs the control group; ^##^ *p* < 0.01 vs the METH group

We selected doses of 1 and 0.25 mg/kg psilocin for injection in the acute METH administration model. We observed significant differences between groups in the acute METH administration model (*F*_(3,21)_ = 15.32, *p* < 0.001) (Figure 2), whereby the activity of the METH group was significantly increased compared to that of the control group (*p* < 0.001). In addition, 1 mg/kg psilocin significantly inhibited METH‐induced hyperactivity in mice, which was statistically significant compared to the METH group (*p* < 0.01). However, 0.25 mg/kg psilocin did not significantly affect METH‐induced hyperactivity.

### 3.3 Regulatory effects of psilocin on the acquisition of METH‐induced CPP

**FIGURE 3:**
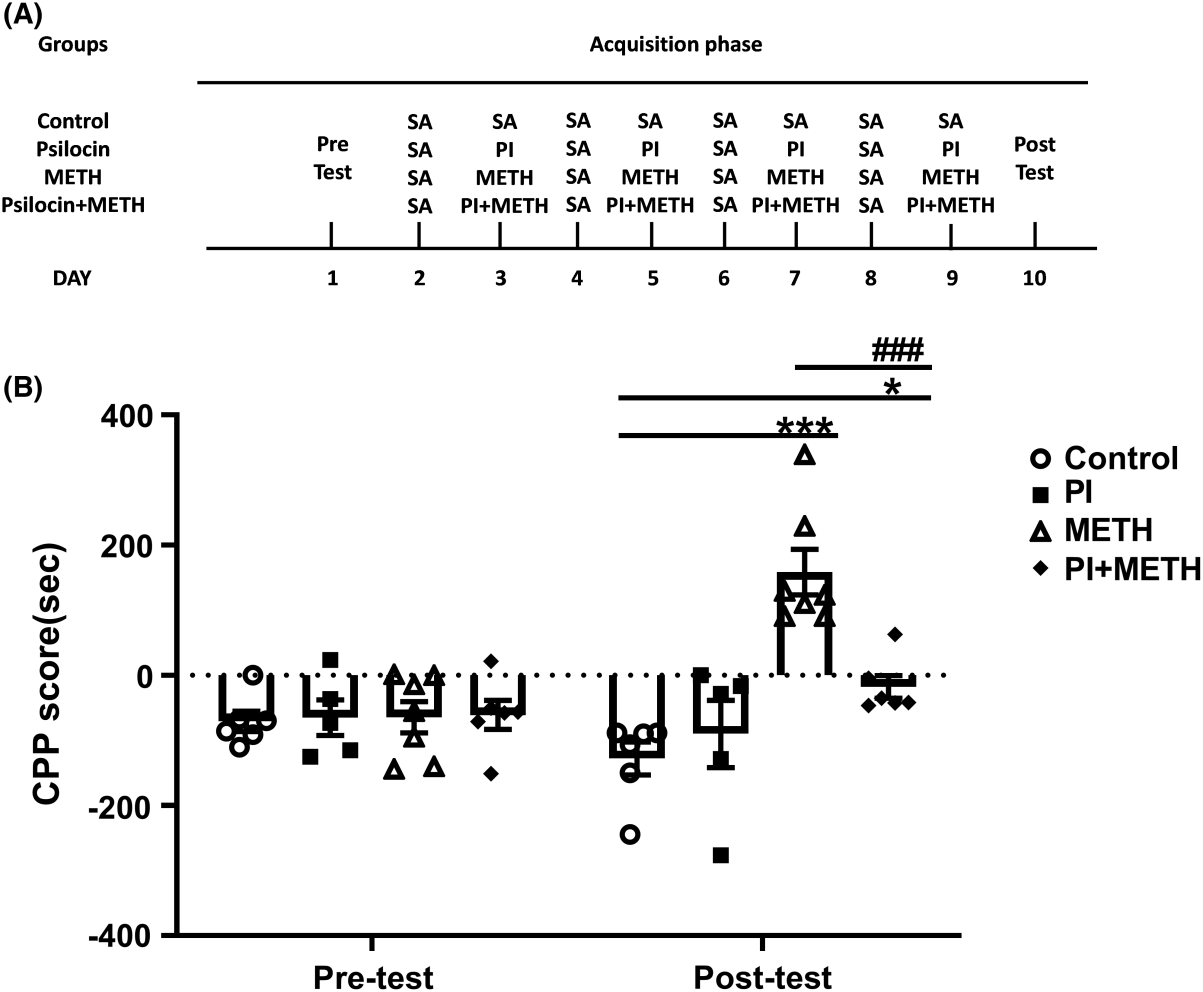
Effects of 1 mg/kg psilocin on the acquisition of METH‐induced CPP. (A) Experimental protocol. (B) Psilocin inhibited CPP acquisition. Data are presented as mean ± SEM (*n* = 5–7). *p < 0.05, ***p < 0.001 vs the control group; ^###^ *p* < 0.001 vs the METH group

A dose of 1 mg/kg psilocin was selected for injection in the METH‐induced CPP model. The procedure of the CPP acquisition phase is presented in Figure 3A. We observed a significant interaction (*F*_(3,20)_ = 8.998, *p* < 0.001), and significant main effects of time (*F*_(1,20)_ = 4.432, *p* = 0.0481) and treatment (*F*_(3,20)_ = 13.34, *p* < 0.001) on the acquisition phase. Post‐test indicated that the CPP scores of the METH group were significantly increased compared to those of the control group (*p* < 0.001). Further, injection of the 1 mg/kg psilocin before METH significantly inhibited the increase in CPP scores of the METH group (*p* < 0.001). CPP scores were higher in the group that received 1 mg/kg psilocin prior to the METH group injection than in the control group (*p* < 0.05). No significant difference in CPP scores was observed between the 1 mg/kg psilocin group and the control group (Figure 3B).

### 3.4 Regulatory effects of psilocin on the extinction of METH‐induced CPP

**FIGURE 4:**
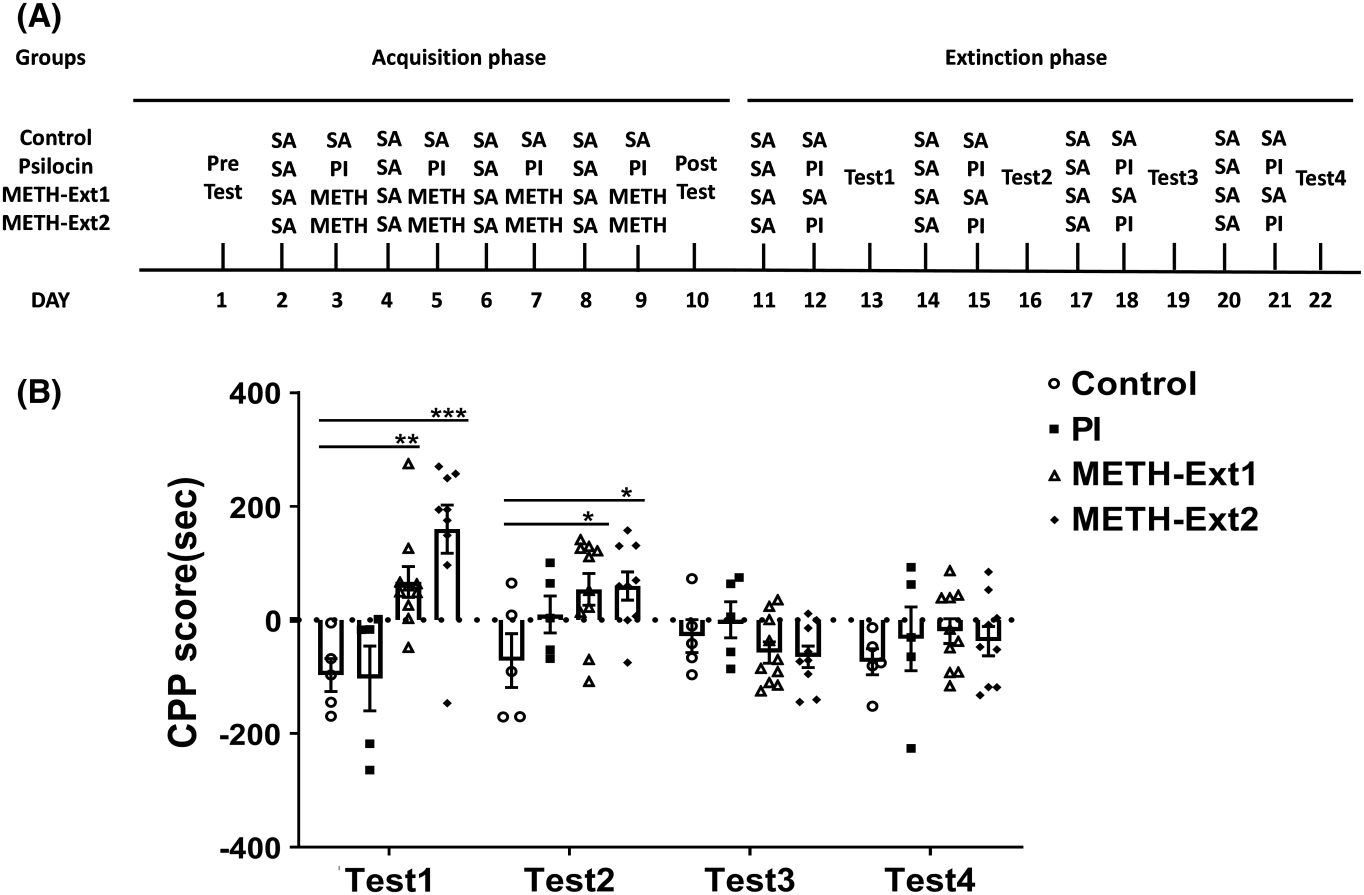
Effects of 1 mg/kg psilocin on the extinction of METH‐induced CPP. (A) Experimental protocol. (B) Psilocin on the extinction of CPP. Data are presented as mean ± SEM (*n* = 5–10). *p < 0.05, **p < 0.01, ***p < 0.001 vs the control group

The procedure of the CPP extinction phase is presented in Figure 4A. In the extinction phase, the METH group was divided into two groups: the METH‐Ext1 group was injected with saline, and METH‐Ext2 group was injected with 1 mg/kg psilocin. The psilocin and control groups underwent continuous training. We observed a significant interaction (*F*_(9,75)_ = 5.177, *p* < 0.001) and significant main effects of time (*F*_(3,75)_ = 3.535, *p* = 0.0187) and treatment (*F*_(3,75)_ = 4.531, *p* = 0.0114) on the extinction phase. No significant differences in CPP score were observed between the psilocin and control groups in each test. The METH‐Ext1 and METH‐Ext2 groups exhibited significant differences in Test 1 (*p* < 0.01 and *p* < 0.001, respectively) and Test 2 (*p* < 0.05 and *p* < 0.05, respectively) compared to the control group (Figure 4B). However, METH‐Ext1 and METH‐Ext2 groups exhibited CPP regression after three extinction training sessions (Figure 4B). No significant differences were observed between the METH‐Ext1 and METH‐Ext2 groups (Figure 4B) in four extinction training sessions.

### 3.5 Regulatory effects of psilocin on the reinstatement of METH‐induced CPP

**FIGURE 5:**
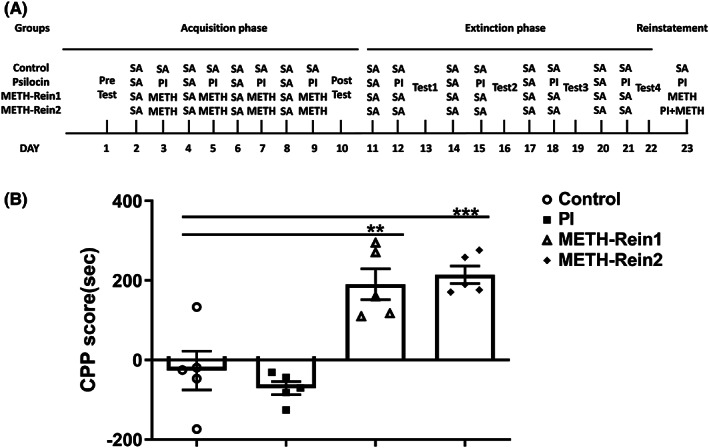
Effects of 1 mg/kg psilocin on the reinstatement of METH‐induced CPP. (A) Experimental protocol. (B) Psilocin on the reinstatement of CPP. Data are presented as mean ± SEM (*n* = 5). **p < 0.01, ***p < 0.001 vs the control group

The procedure of the CPP reinstatement phase is presented in Figure 5A. In the reinstatement phase, the METH‐Ext1 group was divided into METH‐Rein1 and METH‐Rein2 groups. Mice in the METH‐Rein1 group were injected with METH, while mice in the METH‐Rein2 group were injected with 1 mg/kg psilocin 30 min before METH injection. We observed a significant difference in the CPP reinstatement phase (*F*_(3,19)_ = 18.39, *p* < 0.001). CPP scores of the METH‐Rein1 group were significantly increased compared to those of the control group (Figure 5B *, p* < 0.01). CPP scores of the METH‐Rein2 group were significantly increased compared to those of the control group (**Figure** **5** **B** *, p* < 0.001) but were not significantly different from those of the METH‐Rein1 group.

### 3.6 Changes in D2R, p‐AKT, AKT, p‐ERK, and ERK protein levels in the PFC, NAc, and VTA in acute METH administration model

**FIGURE 6:**
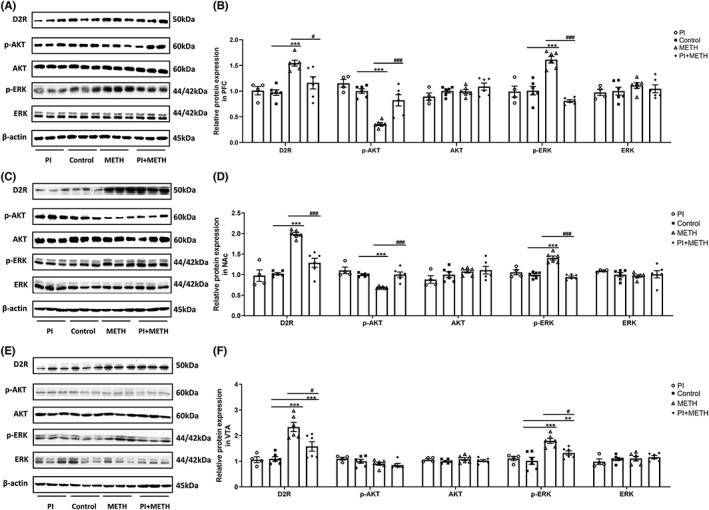
Protein levels of D2R, p‐AKT, AKT, p‐ERK, ERK, and β‐actin in the PFC, NAc, and VTA after acute METH administration. Data are presented as the mean ± SEM (*n* = 4–6). **p < 0.01, ***p < 0.001 vs the control group; ^#^ *p* < 0.05, ^###^ *p* < 0.001 vs the METH group

Expression levels of D2R, p‐AKT, AKT, p‐ERK, and ERK in the PFC, NAc, and VTA were measured. Significant differences were observed in expression levels of D2R (*F*_(3,18)_ = 10.29, *p* < 0.001), p‐AKT (*F*_(3,18)_ = 25.16, *p* < 0.001), and p‐ERK (*F*_(3,18)_ = 26.53, *p* < 0.001) in the PFC. Administration of 1 mg/kg psilocin inhibited the increase in D2R and p‐ERK and ameliorated the decrease in p‐AKT induced by METH (Figure 6A,B). Similarly, significant differences were observed in the expression levels of D2R (*F*_(3,18)_ = 51.88, *p* < 0.001), p‐AKT (*F*_(3,18)_ = 46.30, *p* < 0.001), and p‐ERK (*F*_(3,18)_ = 29.13, *p* < 0.001) in the NAc. Administration of 1 mg/kg psilocin inhibited the increase in D2R and p‐ERK and ameliorated the decrease in p‐AKT induced by METH (Figure 6C,D). Results were observed for the VTA, whereby significant differences were observed in expression levels of D2R (*F*_(3,18)_ = 14.98, *p* < 0.001) and p‐ERK (*F*_(3,18)_ = 11.04, *p* < 0.001) but not of p‐AKT. Notably, administration of 1 mg/kg psilocin inhibited the METH‐induced increase in D2R and p‐ERK (Figure 6E,F). Full unedited blot for Figure 6.

### 3.7 Changes in D2R, p‐AKT, AKT, p‐ERK, and ERK protein levels in the PFC, NAc, and VTA in METH‐induced CPP acquisition

**FIGURE 7:**
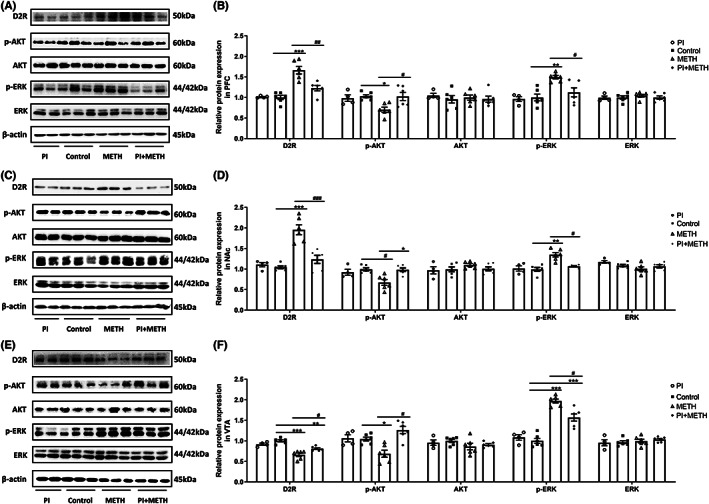
Protein levels of D2R, p‐AKT, AKT, p‐ERK, ERK, and β‐actin in the PFC, NAc, and VTA after the acquisition of METH‐induced CPP. Data are presented as the mean ± SEM (*n* = 4–6). *p < 0.05, **p < 0.01, ***p < 0.001 vs the control group; ^#^ *p* < 0.05, ^##^ *p* < 0.01, ^###^ *p* < 0.001 vs the METH group

Expression levels of D2R, p‐AKT, AKT, p‐ERK, and ERK in the PFC, NAc, and VTA were measured. Significant differences were observed in expression levels of D2R (*F*_(3,18)_ = 21.37, *p* < 0.001), p‐AKT (*F*_(3,18)_ = 4.93, *p* < 0.05), and p‐ERK (*F*_(3,18)_ = 8.90, *p* < 0.001) in the PFC. Administration of 1 mg/kg psilocin inhibited the increase in D2R and p‐ERK expression and ameliorated the decrease in p‐AKT expression induced by METH (Figure 7A,B). Similarly, significant differences were observed in expression levels of D2R (*F*_(3,18)_ = 25.82, *p* < 0.001), p‐AKT (*F*_(3,18)_ = 7.90, *p* < 0.01), and p‐ERK (*F*_(3,18)_ = 13.57, *p* < 0.001) in the NAc. Administration of 1 mg/kg psilocin inhibited the increase in D2R and p‐ERK expression and ameliorated the decrease in p‐AKT induced by METH (Figure 7C,D). Further, significant differences were observed in the expression levels of D2R (*F*_(3,18)_ = 23.08, *p* < 0.001), p‐AKT (*F*_(3,18)_ = 9.78, *p* < 0.001), and p‐ERK (*F*_(3,18)_ = 44.17, *p* < 0.001) in the VTA. Administration of 1 mg/kg psilocin ameliorated the decrease in D2R and p‐AKT expression and inhibited the increase in p‐ERK expression induced by METH (Figure 7E,F). Full unedited blot for Figure 7.

### 3.8 Regulatory effects of TFP‐2HCl and SCH772984 in acute METH administration model

**FIGURE 8:**
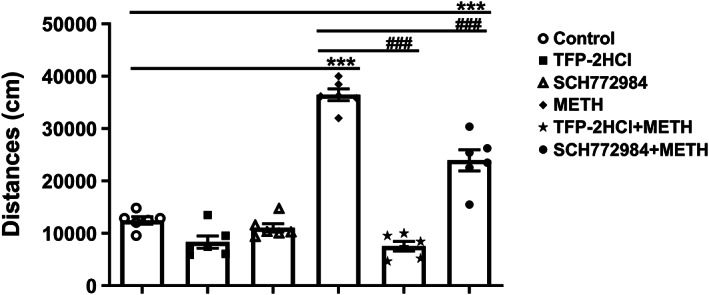
Effects of TFP‐2HCl and SCH772984 on hyperlocomotion induced by acute METH. TFP‐2HCl and SCH772984 could inhibit METH‐induced hyperactivity. Data are presented as mean ± SEM (*n* = 6). ***p < 0.001 vs the control group; ^###^ *p* < 0.001 vs the METH group

In the acute METH administration model, administration of 1 mg/kg psilocin before METH more strongly regulated p‐ERK expression levels than p‐AKT expression levels in the PFC, NAc, and VTA of the METH group. To further investigate the molecular mechanisms of action of psilocin, 2 mg/kg TFP‐2HCl and 10 mg/kg SCH772984 were injected into mice in the acute METH administration model. Mice were divided into six groups (control, TFP‐2HCl, SCH772984, METH, METH + TFP‐2HCl intervention, and METH + SCH772984 intervention groups). We observed significant differences in intervention effects in the acute METH administration model (*F*_(5,30)_ = 90.06, *p* < 0.001) (Figure 8), whereby the activity of the METH group was significantly increased compared to that of the control group (*p* < 0.001). In addition, 2 mg/kg TFP‐2HCl and 10 mg/kg SCH772984 significantly inhibited METH‐induced hyperactivity in mice compared to that in the METH group (both *p* < 0.001). No significant differences were observed between the 2 mg/kg TFP‐2HCl, 10 mg/kg SCH772984, and control groups.

### 3.9 Regulatory effects of TFP‐2HCl and SCH772984 on the acquisition of METH‐induced CPP

**FIGURE 9:**
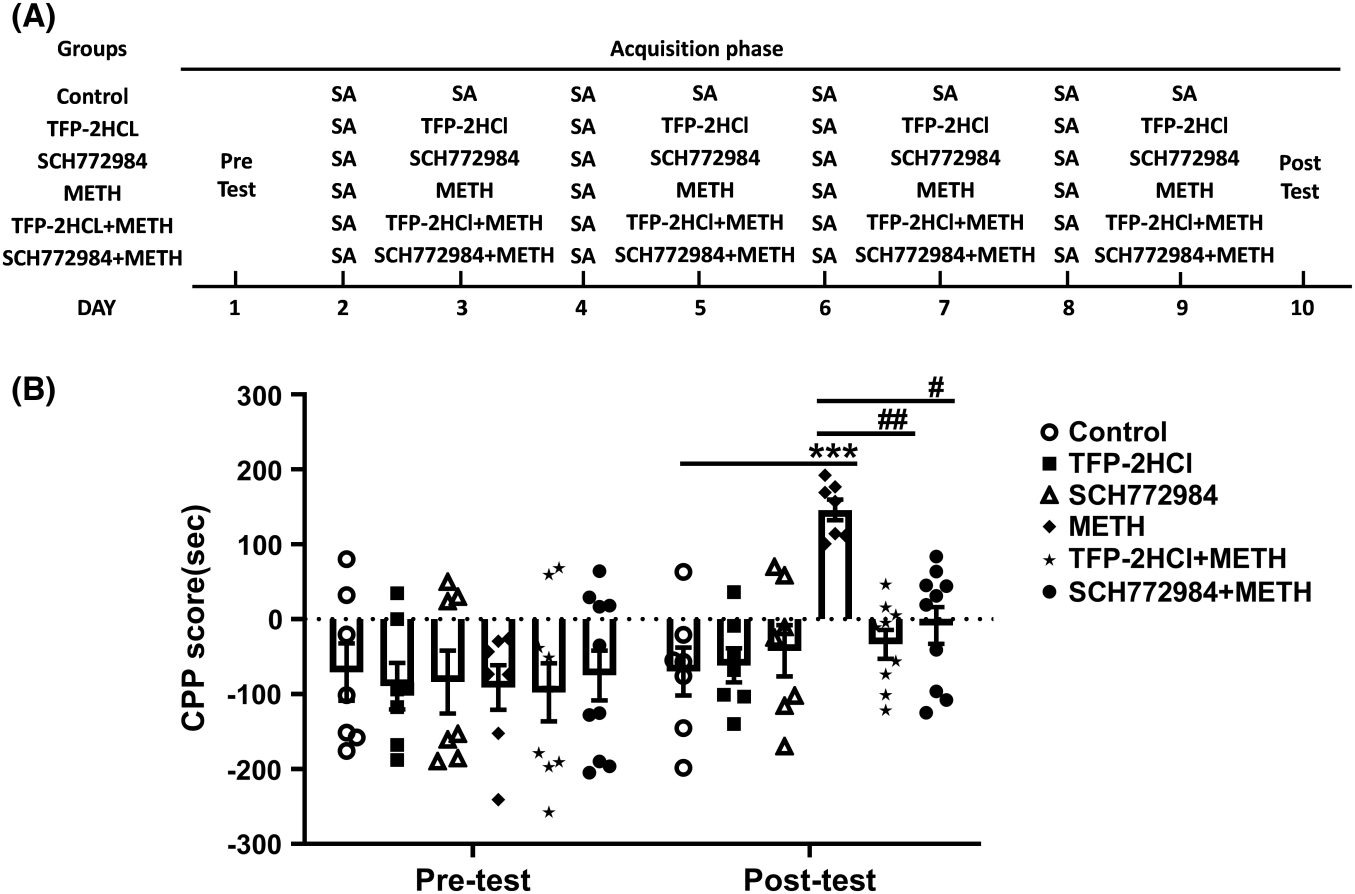
Effects of TFP‐2HCl and SCH772984 on the acquisition of METH‐induced CPP. (A) Experimental protocol. (B) TFP‐2HCl and SCH772984 inhibited CPP acquisition. Data are presented as mean ± SEM (*n* = 7–10). ***p < 0.001 vs the control group; ^#^ *p* < 0.05, ^##^ *p* < 0.01 vs the METH group

With regard to the acquisition of METH‐induced CPP, administration of 1 mg/kg psilocin before METH more strongly regulated p‐ERK expression levels than p‐AKT expression levels in the PFC, NAc, and VTA of the METH group. To further investigate the molecular mechanisms of action of psilocin, 2 mg/kg TFP‐2HCl and 10 mg/kg SCH772984 were injected into mice in the acquisition of the METH‐induced CPP model. The procedure of the CPP acquisition phase is presented in Figure 9A. We observed a significant interaction (*F*_(5,41)_ = 3.135, *p* < 0.05) and significant main effects of time (*F*_(1,41)_ = 15.53, *p* < 0.001) and treatment (*F*_(5,41)_ = 2.917, *p* < 0.05) on the acquisition phase. Post‐test indicated that the CPP score of the METH group was significantly increased compared to that of the control group (*p* < 0.001). Moreover, administration of 2 mg/kg TFP‐2HCl and 10 mg/kg SCH772984 before METH significantly inhibited the increase in CPP score of the METH group (*p* < 0.01 and *p* < 0.05, respectively). CPP scores of the groups injected with 2 mg/kg TFP‐2HCl and 10 mg/kg SCH772984 prior to METH were not significantly different from those of the control group (Figure 9B).

## 4 DISCUSSION

Previous studies have indicated that the range of 0.25–4 mg/kg psilocin affects the behavior of rats in the open field, carrousel maze, and Morris water maze.^22^ Therefore, in this study, psilocin was administered at three different concentrations (5, 1, and 0.25 mg/kg). A high dose of psilocin (5 mg/kg) promotes activity in mice, whereas 1 and 0.25 mg/kg of psilocin exert minimal effects on activity in mice. In previous studies, a higher dose (4 mg/kg) of psilocin affected locomotor,^22^ however, a lower psilocin dose (1 mg/kg) did not significantly affect locomotor behavior relative to that of control. Indeed, the physical and psychological dependence potential of magic mushrooms has been reported to be low.^23^

METH induces behaviors such as hyperlocomotion, CPP, and self‐administration.^24^ Neuroimaging studies have demonstrated that METH causes cortical and striatal abnormalities that induce addiction‐related phenotypes, thereby promoting compulsive drug use.^25^ In this study, 1 mg/kg psilocin counteracted METH‐induced elevation of activity. Moreover, in the acquisition phase, 1 mg/kg psilocin inhibited CPP formation. This study highlights the potential of psilocin for the treatment of acute and chronic METH‐induced alterations of behavior. Psilocin is a postsynaptic 5‐HTR2A agonist.^6^ Previous research has demonstrated that direct infusion of 5‐HT inhibited DA activity and reduced DA‐mediated functions including spontaneous locomotor activity, amphetamine‐induced locomotor activity and stereotypies, turning behavior, and extra‐pyramidal functions.^26^ Psilocin and psilocybin have recently received growing attention in addiction research.^27^ For example, a recent study reported that psilocin significantly reduced the percentage of drinking and heavy drinking days in alcohol‐dependent patients.^28^ Crucially, after treatment with psilocybin, 80% of tobacco‐dependent patients had quit smoking after 6 months.^5^

Recent studies have reported the existence of D2R‐5‐HT2AR heteromers in cellular models whereby the ic3 region plays a key role in the dimerization of 5‐HT2AR and D2R.^8^ Moreover, there is an opposing action of 5‐HT on DA in the nigrostriatal system.^29^ For instance, decreasing the density of 5‐HT transporters results in a disinhibition of the mesolimbic DA system and inhibitory effects of 5‐HT2 mediated by the local dendritic release of DA which promotes D2R activation.^30^ In this study, we examined D2R expression given that previous literature has demonstrated that cocaine‐ and heroin‐seeking behaviors were strongly associated with selective stimulation of D2Rs.^31^ Moreover, experimental evidence indicates that D2R‐expressing neurons are critical for motor behavioral control.^32^ The current findings indicated that in the acute METH administration model, D2R expression was increased in PFC, NAc, and VTA in the METH group. During the acquisition phase of CPP, D2R expression in PFC and NAc was significantly increased in the METH group. Similarly, METH increased D2R expression associated with activation of dopaminergic neurons in the mesolimbic pathway.^13^ Conversely, the level of D2R expression in the VTA was decreased in the METH group. Previous studies have illustrated a biphasic relationship between METH and D2R. Long‐term overdose has been associated with decreased expression and sensitivity of D2R.^33^ Research has indicated that METH exerts direct neurotoxic effects on D2R due to METH‐induced hyperthermic responses.^10^ Moreover, AMPH self‐administration reduces D2Rs in the VTA which is more susceptible to changes compared to the NAC, while significant changes in the NAC may require prolonged exposure to AMPH.^34^ Of note, we observed that in the METH acute administration model, 1 mg/kg psilocin reduced the METH‐induced increase in D2R expression in the PFC, NAc, and VTA. Moreover, in the acquisition phase of CPP, administration of 1 mg/kg psilocin before METH significantly reduced the METH‐induced increase in D2R expression in the PFC and NAc. A recent study reported that 5‐HTR agonists induced allosteric inhibition of D2R signaling, and the D2R protomer was reduced by 5‐HT‐induced activation of the 5‐HT2AR protomer.^35^

ERK1/2 and AKT activation induces alterations in striatal DA‐dependent behaviors. Previous studies have indicated that D2R activation results in increased phosphorylation of ERK1/2, and over‐activation of D2Rs leads to a decrease in AKT phosphorylation in cortical neurons.^16^, ^36^ The results indicated that the expression of p‐AKT in VTA was not significantly altered in the METH acute administration model, while METH decreased the expression of p‐AKT in the VTA during the CPP acquisition period. Similarly, studies have reported a decrease in AKT signaling in the VTA during chronic morphine.^37^ In addition, both in METH acute administration model and in the acquisition phase of CPP 1 mg/kg psilocin injected before METH could reverse regulate the level of p‐ERK expression more significant than p‐AKT in the PFC, NAc and VTA of the METH group. In addition, in both the METH acute administration model and acquisition phase of CPP, administration of 1 mg/kg psilocin before METH more strongly regulated the expression level of p‐ERK than that of p‐AKT in the PFC, NAc, and VTA in the METH group. Based on its pharmacological characteristics, the p‐ERK assay provides a robust readout of D2R activation and has a medium throughput, which may effectively screen many compounds.^20^ Moreover, Akt dephosphorylation is a late biochemical reaction stimulated by DA receptors,^21^ and prolonged stimulation of D2‐class receptors leads to specific dephosphorylation of AKT on its regulatory Thr308.^38^

In the present study, we observed that the inhibitory effects of psilocin on METH‐induced expression of D2R and p‐ERK were potentially associated with the blocking effect of psilocin on METH‐induced hyperactivity and acquisition of METH‐induced CPP. To determine the role of D2R and p‐ERK in METH‐induced hyperactivity and acquisition of METH‐induced CPP, we examined behavioral changes in mice after administering trifluoperazine 2HCl (TFP‐2HCl) and SCH772984 to inhibit D2R and p‐ERK, respectively. TFP‐2HCl is a phenothiazine derivative that exerts specific actions in the brain by inhibiting postsynaptic D2Rs. Moreover, trifluoperazine blocks postsynaptic D2Rs in midbrain limbic and cortical projections.^39^ Reports have demonstrated that 2–3 mg/kg TFP‐2HCl affects the central nervous system in mice.^40^, ^41^, ^42^ SCH772984 is a selective ERK1/2 inhibitor that binds to unphosphorylated and inactive ERK1/2 and effectively inhibits ERK1/2 phosphorylation.^43^ Previous research has reported that the effective dose range of SCH772984 in mouse models is 10–20 mg/kg.^44^, ^45^, ^46^

In conclusion, 1 mg/kg psilocin counteracted METH‐induced hyperlocomotion in an acute METH administration model and inhibited METH‐induced CPP formation. In addition, the regulatory effect of psilocin on METH were underscored by altered expression of D2R and p‐ERK in PFC, NAc, and VTA. Injection of 2 mg/kg TFP‐2HCl and 10 mg/kg SCH772984 prior to METH administration blocked METH‐induced hyperactivity and acquisition of METH‐induced CPP, indicating that the D2R and p‐ERK play a key role in METH‐induced alterations of behavior. Our findings suggest that psilocin may block METH‐induced alterations of behavior in mice via D2R‐mediated signal regulation of ERK phosphorylation.

## AUTHOR CONTRIBUTIONS

JW, XSL, and TL conceived and designed the experiments. JW, ML, and QS performed the experiments. HYQ, RA, and GJS assisted with the data analysis. JW wrote the manuscript. HL provided revisions to the manuscript. All authors contributed to and approved the final manuscript.

## CONFLICT OF INTEREST

We have no conflicts of interest to declare.

## Data Availability

All data included in this study are available from the corresponding author upon reasonable request.
